# Supplementary material for: Pixel-wise programmability enables dynamic high-SNR cameras for high-speed microscopy
Source: Nat Commun. 2024 May 27;15:4480. doi: 10.1038/s41467-024-48765-5 (PMC11530699; doi:10.1038/s41467-024-48765-5)
Supplement: Supplementary file 1 — Supplementary Information [file 41467_2024_48765_MOESM1_ESM.pdf]

Supplementary Figures

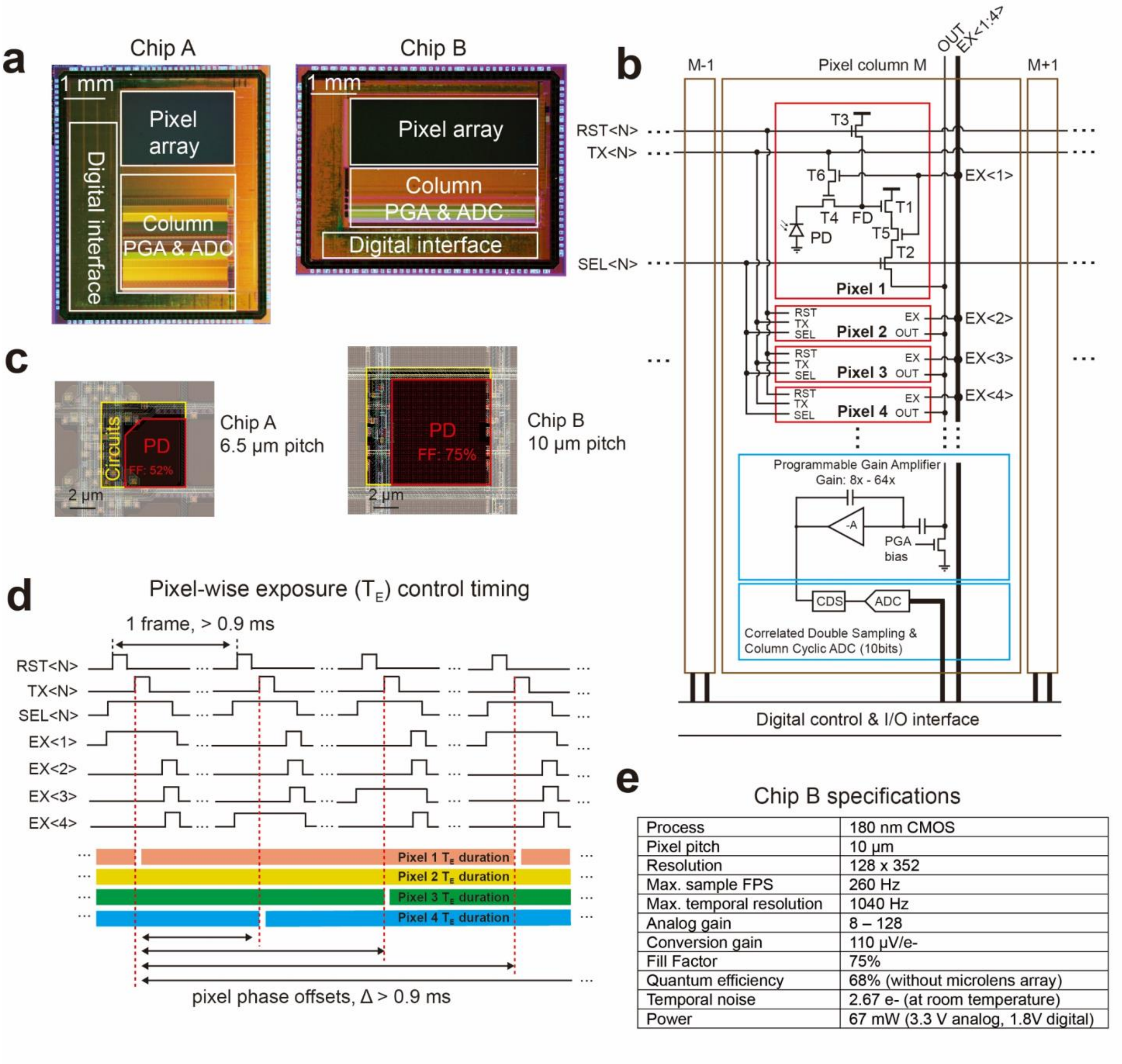

Process	180 nm CMOS
Pixel pitch	10 µm
Resolution	128 x 352
Max. sample FPS	260 Hz
Max. temporal resolution	1040 Hz
Analog gain	8 – 128
Conversion gain	110 µV/e-
Fill Factor	75%
Quantum efficiency	68% (without microlens array)
Temporal noise	2.67 e- (at room temperature)
Power	67 mW (3.3 V analog, 1.8V digital)

Supplementary Figure 1: PE-CMOS design detail.
a, Micrograpy of two PE-CMOS prototypes implemented in a 180 nm CMOS image sensor process. Chip A and B feature 6.5 and 10 µm pixels, respectively.
b, PE-CMOS circuits schematic for Chip B. Pixels are organized in pixel groups (PG) consisting of K row and 1 column (K = 8 in Chip A, 4 in Chip B). Each column shares one amplifier and ADC. The pixel design features 6 transistors (T1 – T6) to provide pixel-wise exposure control while maximizing the pixel fill factor.
c, Pixel layouts (PD: photodiode, FF: photodiode fill factor).
d, Example timing control for programmable pixel-wise exposure. A pixel only ends the exposure when its corresponding TX, SEL, and EX signal are high. Its exposure continues otherwise. Pixel-wise exposure control can be achieved by programming column-wise signal EX in coordination with row-wise signals (TX, SEL and EX).
e, sensor specifications table.

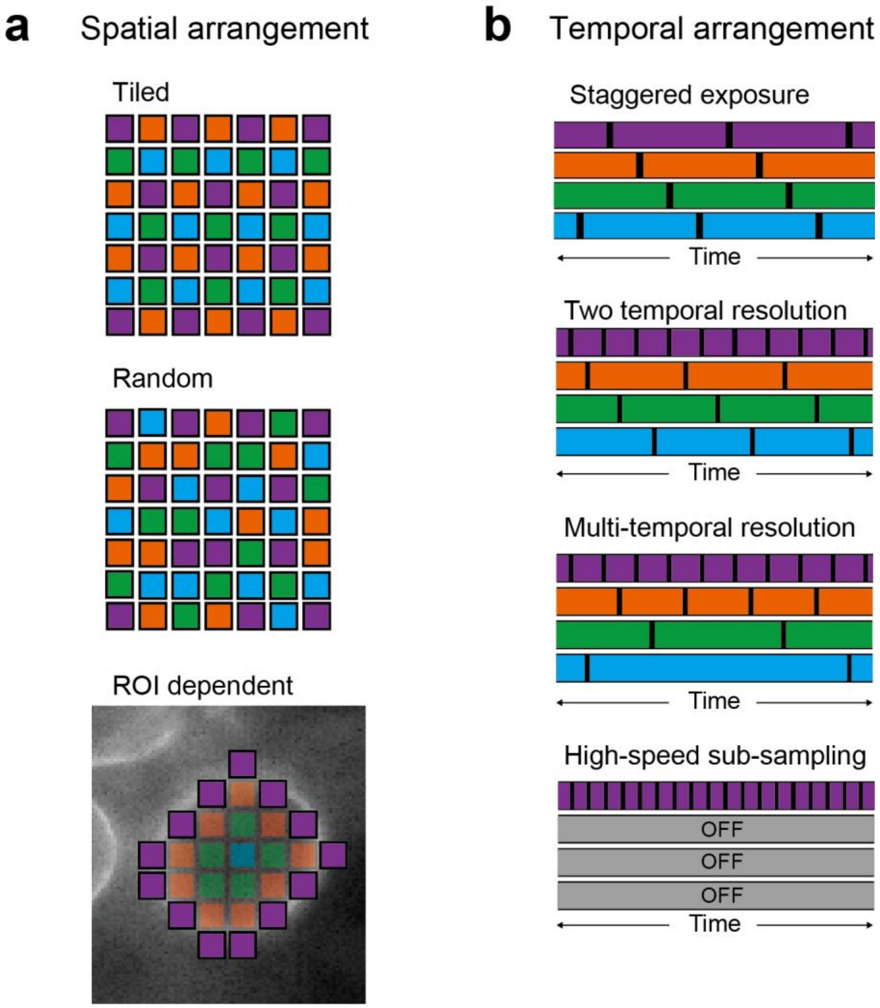

Supplementary Figure 2: Examples of pixel sampling patterns enabled by the PE-CMOS
a, spatial arrangement examples. Pixels of the same color have identical exposure duration (T_E) and phase offsets (Δ). In addition to a tiled or random arrangement, pixels can be arranged according to the spatial features of the biological scene depending on the ROI. Pixels at the bright regions of the cell membrane are configured to have short T_E with a high sampling rate, while pixels in the dim regions use long T_E with a slow sampling rate to enhance the SNR.
b, Temporal arrangement example, where pixels' relative phase and duration are programmable.

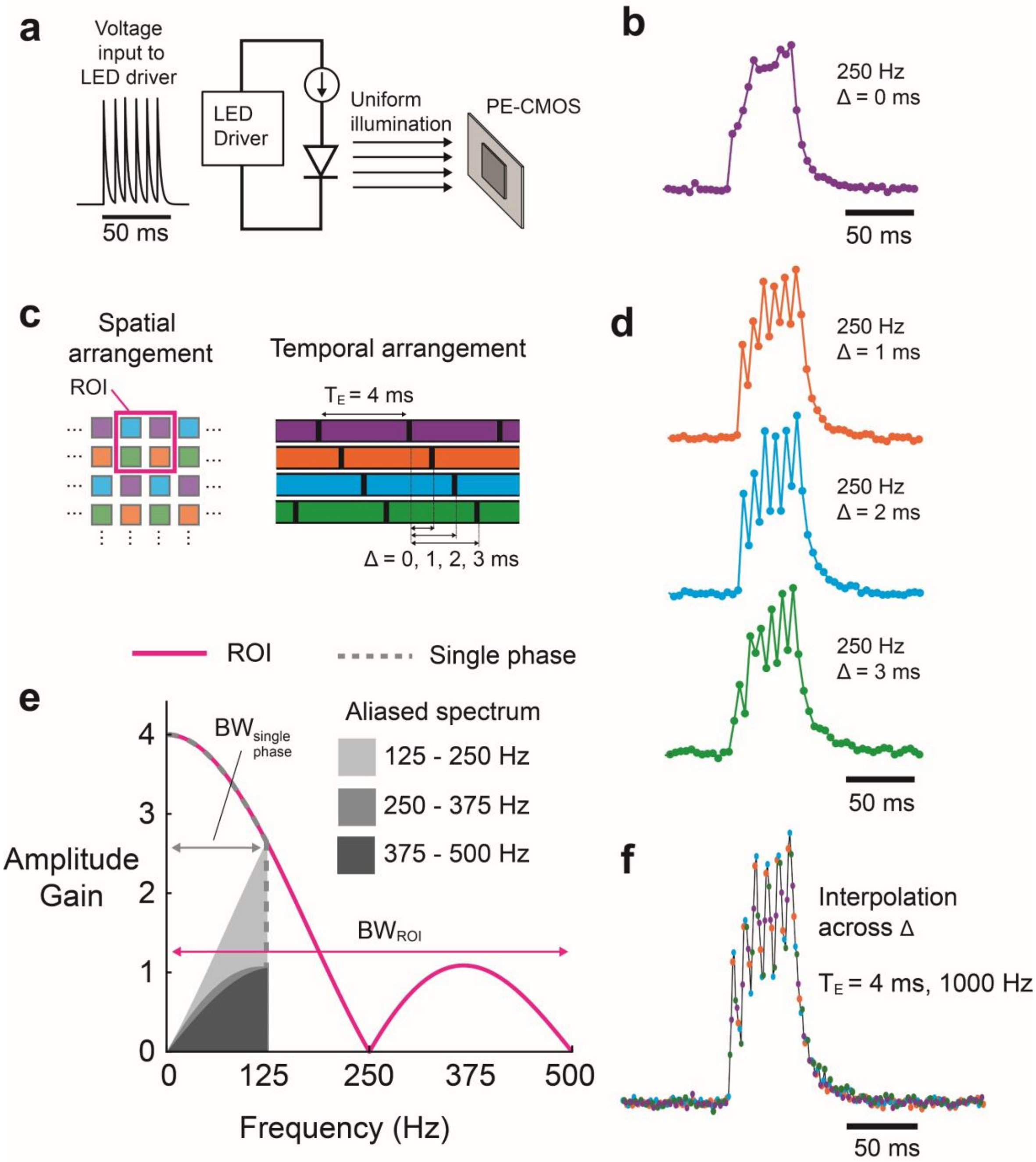

Supplementary Figure 3: A benchtop example showing phase offset pixels can improve ROI temporal resolution

a, a test setup using an LED to illuminate the PE-CMOS sensor uniformly. The LED is driven with a current mimicking GEVI spike burst events (6 spikes, 8 ms inter-spike interval).

b, pixels sampling at 250 Hz with exposure (T_E) of 4 ms without phase offset (Δ = 0 ms) are unable to resolve individual spikes due to the slow sampling rate (purple trace).

c, PE-CMOS sampling configuration with various phase offset pixels. The ROI consists of pixels with phase offsets Δ of 0, 1, 2, and 3 ms (color traces).

d, PE-CMOS pixels at different phases together samples the fast-spiking activity that is unsolvable using a single phase.

e, the frequency response of pixels without phase offsets vs. pixels with phase offsets. In this example, the single phase's bandwidth is 125 Hz, with distortion added by frequency aliasing (gray color). With phase offset, PE-CMOS extends the bandwidth of the ROI by 4 times (500 Hz) and avoids aliasing effects (magenta trace).

f, an 1000 Hz equivalent high-speed time series (8 samples between two consecutive spike peaks) from interpolating PE-CMOS pixels at different phases.

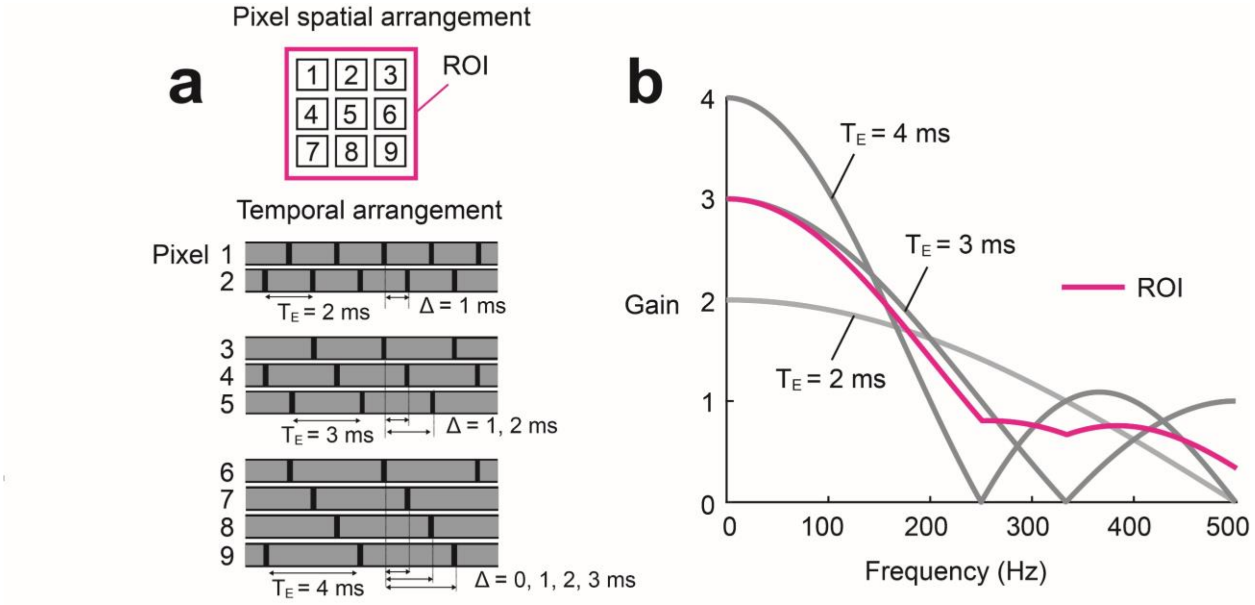

Supplementary Figure 4: PE-CMOS frequency response of ROI containing multiple pixel exposure.
a, An ROI with 9 pixel configured with T_E = 2, 3, and 4 ms and phase offsets of Δ = 0, 1, 2, and 3 ms.
b, The combined ROI's frequency response eliminates narrow band attenuations at different T_E 's frequenecy response.

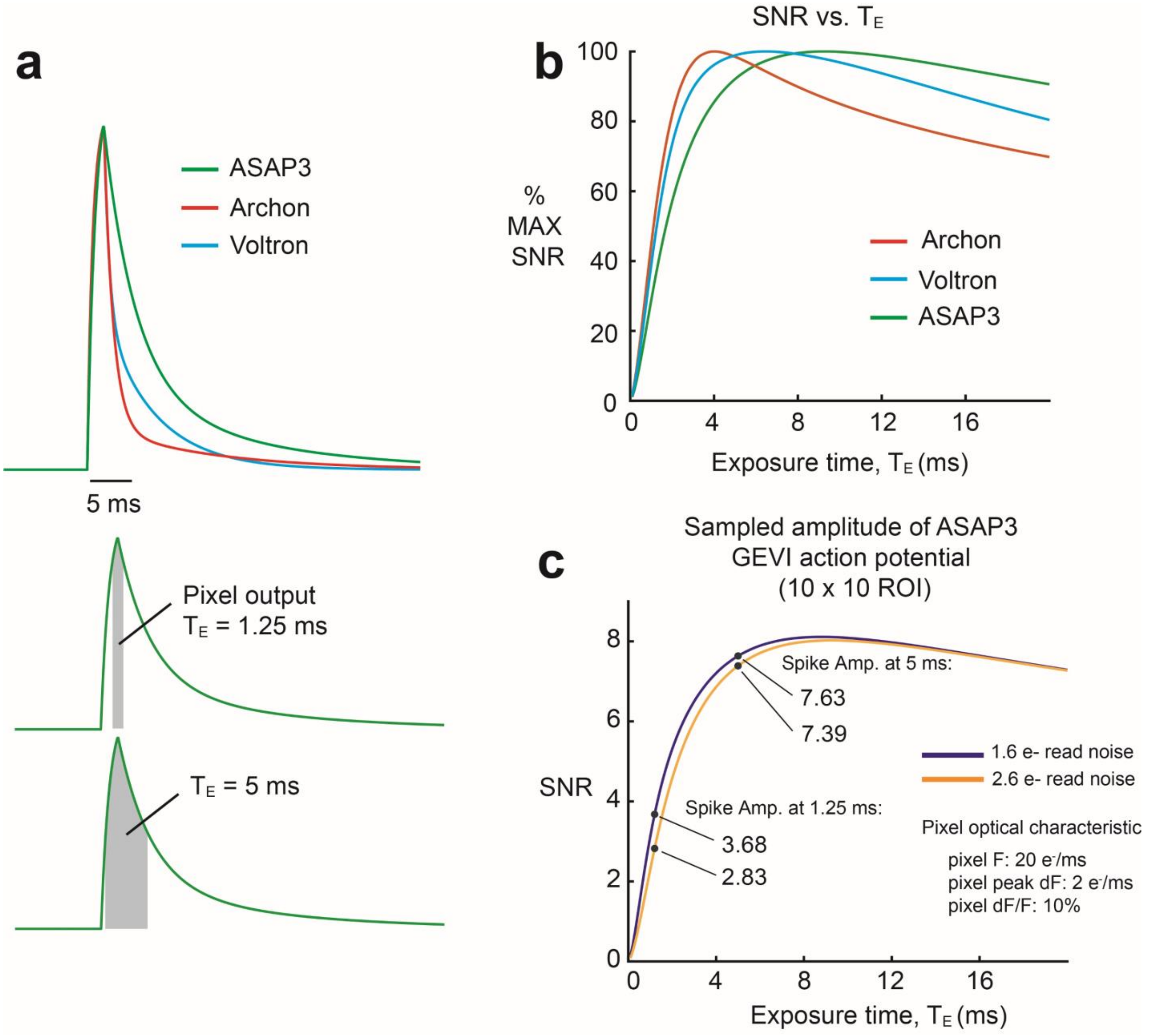

Supplementary Figure 5: Modeling the relationship between pixel exposure and SNR of sampling GEVI action potentials

a, Spikes modeled using timing parameters of several GEVI indicators. A pixel integrates fluorescence photons for exposure time, T_E, which equals to the area under the curve.

b, Pixel SNR with respect to exposure time, T_E, for spikes modeled in panel A. Each curve is normalized with the maximum SNR set to 100%. The SNR increases fast with longer T_E but drops gradually as extra integration time adds more shot noise than signal power due to baseline fluorescent.

c, SNR curves at sampling the ASAP3 spikes. Two curves showing the SNR of pixels with different read noise (ROI: 10 x 10 pixels). Despite the PE-CMOS (orange curve) having slightly higher read noise of 2.6 electrons (e^-) rms than the sCMOS (blue curve, 1.6 e^- rms), longer T_E (5 ms) still enables the PE-CMOS to have >2 times better SNR than the sCMOS with shorter T_E (1.25 ms).

.

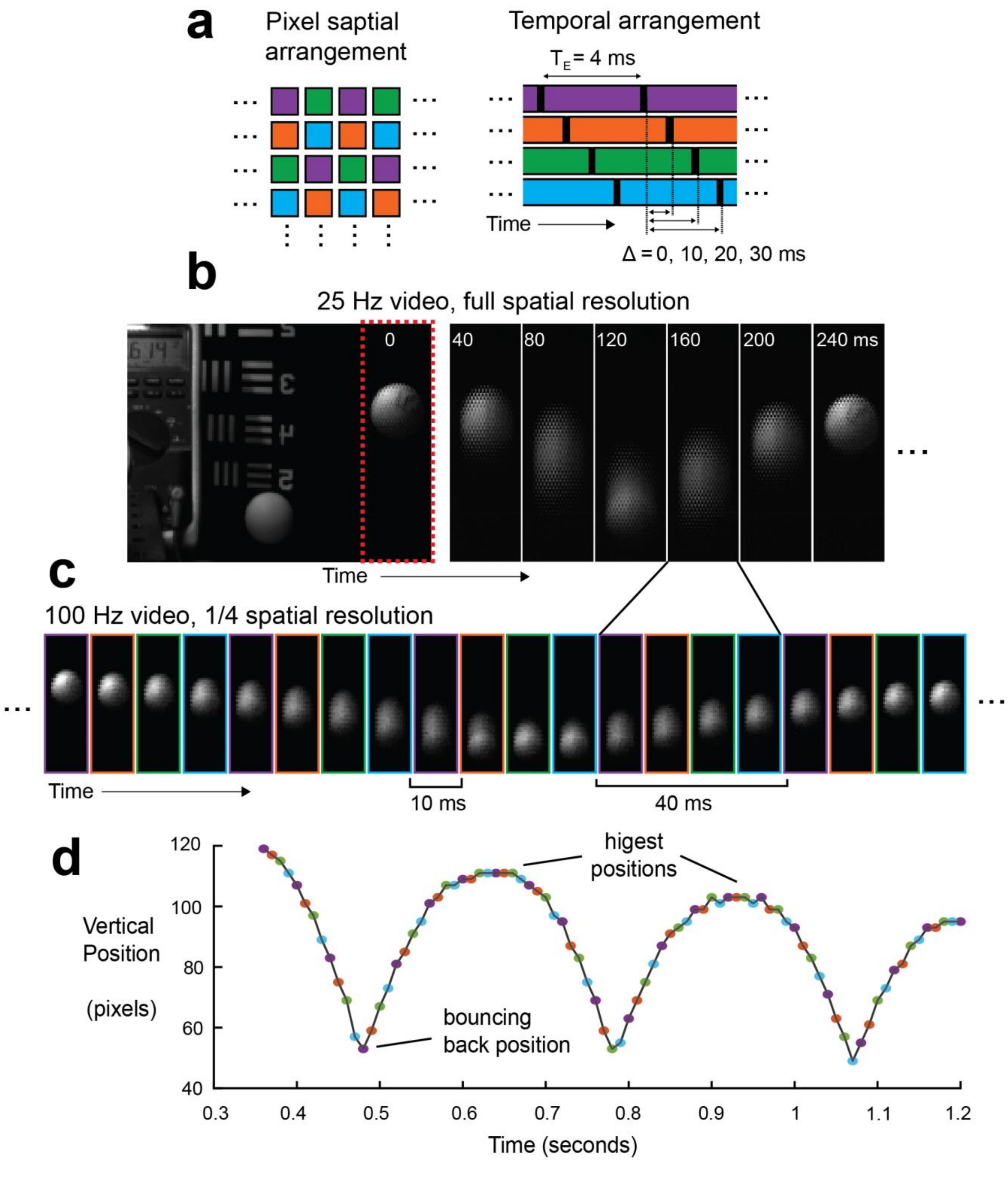

Supplementary Figure 6: Applying the PE-CMOS to general imaging application to resolve fast motion with low sampling rate.

a, a staggered pixel configuration with exposure (T_E) of 40 ms and phase offset (Δ) = 0, 10, 20, 30 ms. Pixel of the same color has identical exposure and phase offset.

b, A single frame at full spatial resolution from a video at 25 Hz speed and seven frames taken from a 25 Hz video of the section highlighted by the dotted red line. The bouncing ball's motion is embedded within the spatial patterns at full each.

c, splitting each full-resolution frame into four sub-frames, each with 1/4 resolution to resolve a 100 Hz equivalent video.

d, Resolving the position of the bouncing ball at 100 Hz resolution while each pixel is sampled at 25 Hz, results in 4-fold gain in temporal resolution by using phase-shifted pixels.

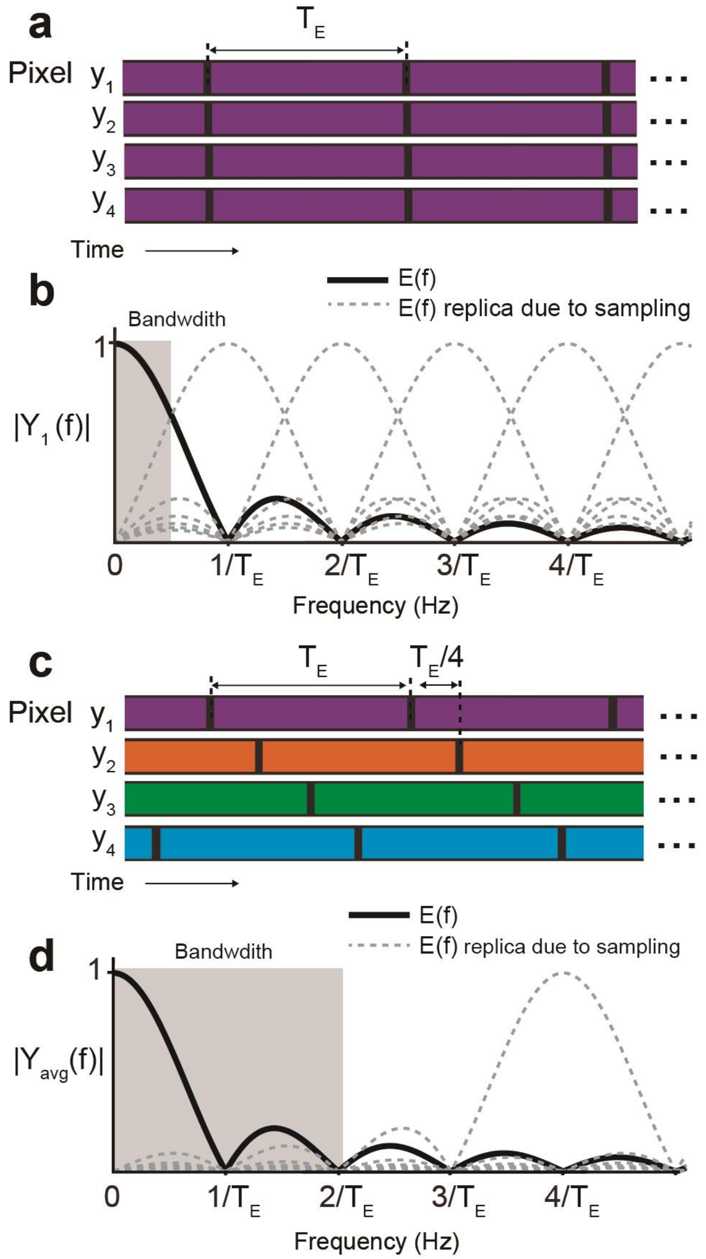

Supplementary Figure 7. Multi-phase sampling enhances the temporal resolution without increasing sampling speed

A. Conventional camera samples all the pixels ($y_1,\ldots,y_4$) concurrently (global shutter) or in fast line sequences (rolling shutter). With pixel exposure of T_E and sampling rate at $1/T_E$.

B. Frequency spectrum of the averaged pixel value, y_{avg}. Its Nyquist bandwidth is limited to $1/(2T_E)$ and suffers from a significant amount of temporal aliasing.

C. In multi-phase sampling, pixels of exposure T_E are phase-offsetted in multiples of $T_E/4$.

D. Without increasing the sampling rate, multi-phase exposures extend the y_{avg} bandwidth by four times to $2/T_E$ and mitigate temporal aliasing by pushing the replica spectra to higher frequencies.

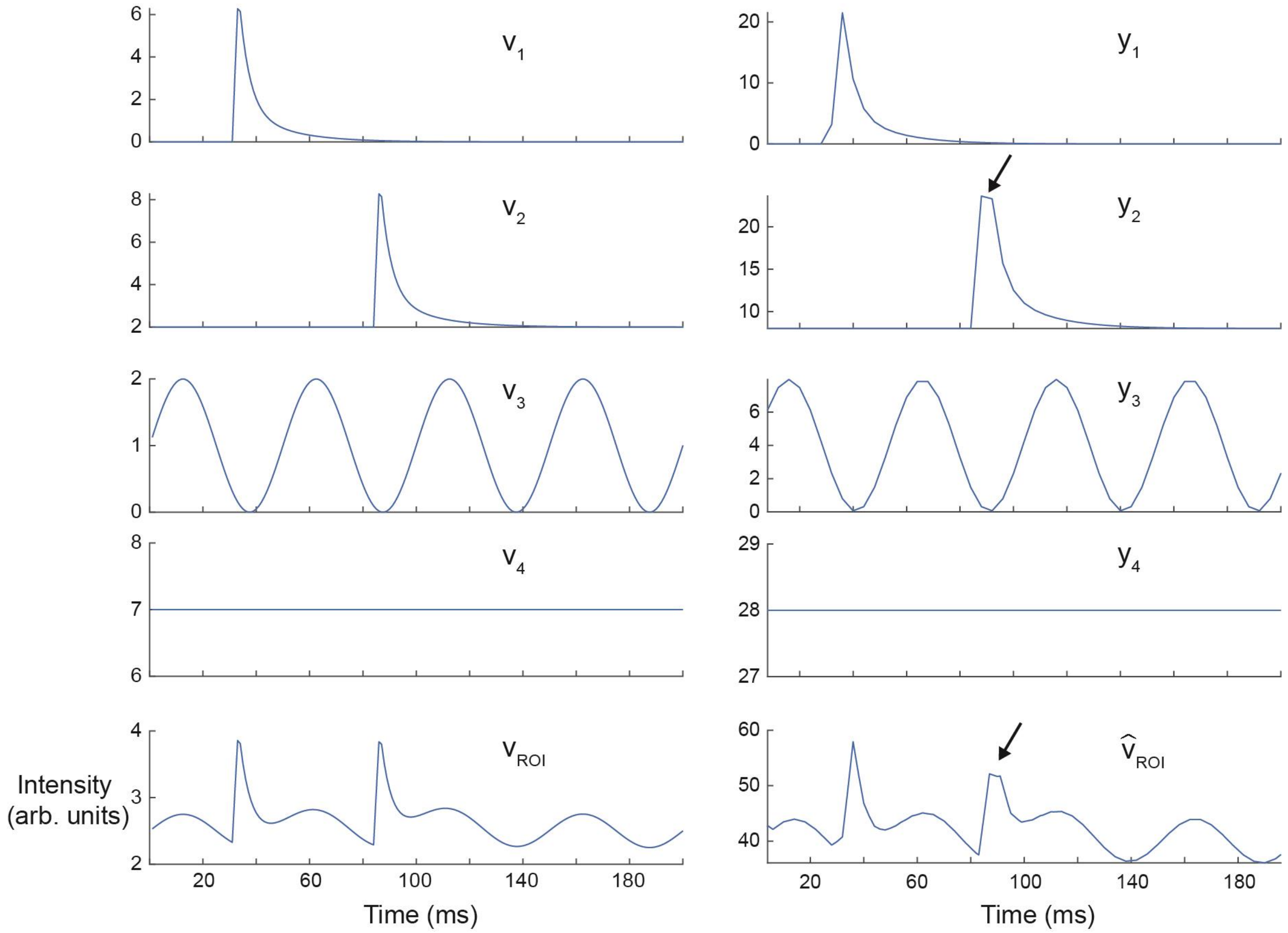

Supplementary Figure 8. Example of interpolating the PE-CMOS pixels with uncorrelated signal. The time series of 4 pixels, $\boldsymbol{v}_1 \ldots \boldsymbol{v}_4$, contains uncorrelated signal, with $\boldsymbol{v}_{ROI}$ representing the average of these signals. We mimic the exposure and phase-shifted sampling of these pixels to get $\boldsymbol{y}_1 \ldots \boldsymbol{y}_4$. We do this by convolving phase-shifted version of $\boldsymbol{v}_1 \ldots \boldsymbol{v}_4$ with a box function of length 4, followed by 4x down-sampling. We can see the effect down-sampling aliases the high-frequency part of the signal of $\boldsymbol{y}_2$, especially at the peak of the spike (black arrow).

We can apply our interpolation outlined in Eq. 7, which results in $\hat{\boldsymbol{v}}_{\boldsymbol{ROI}}$. We can see that $\hat{\boldsymbol{v}}_{\boldsymbol{ROI}}$ approximates $\boldsymbol{v}_{ROI}$, but underfits the spike at the location pointed by the black arrow. This is because we only sampled $\boldsymbol{y}_2$ at a single phase since $\boldsymbol{y}_2$ is uncorrelated with other pixels.
